# Phase-Specific Parameter Estimation in Chiral HPLC Using 1‑Site, 2‑Site Stochastic Models, and Unified Equation Approach

**DOI:** 10.1021/acs.analchem.6c01634

**Published:** 2026-04-29

**Authors:** Arash Mirzahosseini, Ali Mhammad, Gergely Dombi, Balázs Balogh, Annamária Sepsey, Tamás Pálla, Simon Horváth, Eliza Tóth, Zoltán-István Szabó, Gábor Németh, Attila Felinger, Oliver Trapp, Gergő Tóth

**Affiliations:** † Department of Pharmaceutical Chemistry, 37637 Semmelweis University, Budapest H-1092, Hungary; ‡ Center for Pharmacology and Drug Research & Development, Semmelweis University, Budapest H-1085, Hungary; § Department of Organic Chemistry, Semmelweis University, Budapest H-1092, Hungary; ∥ Institute of Bioanalysis, Medical School, 37656 University of Pécs, Pécs H-7624, Hungary; ⊥ Drug Substance Analytical Development Division, 58965 Egis Pharmaceuticals Plc, Budapest H-1106, Hungary; # Department of Pharmaceutical Industry and Management, George Emil Palade University of Medicine, Pharmacy, Science and Technology of Targu Mures, Targu Mures 540142, Romania; ∇ Sz-imfidum Ltd., Covasna 525401, Romania; ○ Department of Analytical and Environmental Chemistry and Szentágothai Research Center, University of Pécs, Pécs H-7624, Hungary; ◆ Department of Chemistry, 9183 Ludwig-Maximilians-Universität München, München D-81377, Germany

## Abstract

On-column enantiomerization remains a challenge for quantitative chiral analysis, but it also provides an opportunity to extract mechanistic information directly from chromatographic data. Here, we present an extended automated workflow in R language for statistical computing for modeling dynamic chromatographic profiles (Batman peaks) of interconverting enantiomers, incorporating a two-site stochastic formulation that distinguishes nonselective and enantioselective interactions on cellulose-based chiral stationary phases. Overload experiments were analyzed using a competitive bi-Langmuir isotherm to estimate site ratios and affinities, which were subsequently integrated into stochastic peak-shape analysis to obtain forward and reverse interconversion rate constants across multiple eluents, temperatures, and flow rates. To decompose these apparent rates into mobile and stationary phase contributions, we developed a mixed-effect model based on capacity factor linearization, followed by empirical Bayesian inference. Mobile phase-specific rate constants exhibited consistent Eyring–Polányi behavior and showed excellent agreement with independently measured off-column circular dichroism kinetics. The results demonstrate that detailed kinetic and thermodynamic characterization of enantiomerization can be obtained directly from routine liquid chromatography measurements. The proposed workflow delivers an integrated, fully automated platform that enhances the analytical utility of dynamic chiral separations and is broadly applicable to systems exhibiting on-column interconversion.

## Graphical abstract

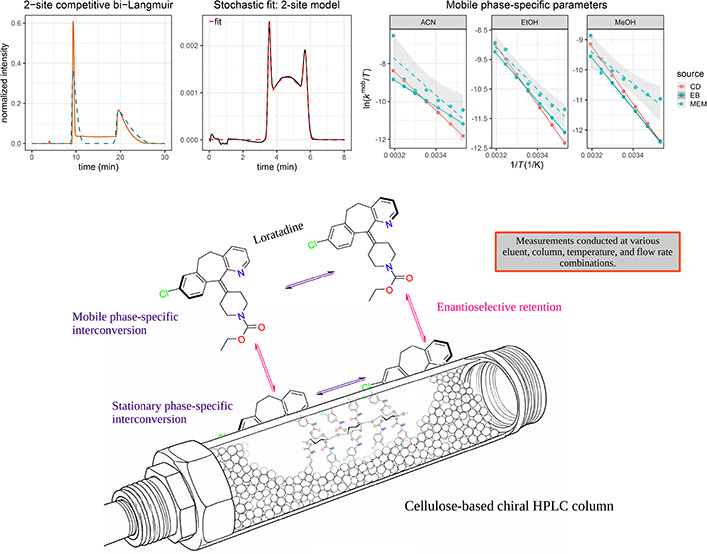

## Introduction

Enantiomers are ubiquitous in nature, and their individual forms often exhibit markedly different biological, pharmacological, and toxicological profiles. The ability to isolate, quantify, and characterize both enantiomers is therefore essential in drug development and regulation.^1^ Differences in receptor binding, metabolism, and clearance make chiral analysis a fundamental component of pharmaceutical science. Enantiomer interconversion represents an additional layer of complexity, as interconverting systems may display altered *in vivo* half-lives or unexpected pharmacokinetic behavior.^2^ Measuring these interconversion rates is crucial to understanding dynamic chiral systems under physiological conditions. Chromatographic approaches remain particularly attractive because they provide a direct experimental window into the kinetic processes occurring between the enantiomers.

On-column interconversion during chiral HPLC (high-performance liquid chromatography) has long been recognized and studied, manifesting in “Batman-shaped” peaks. This characteristic coalescence phenomenon reflects the interplay between chromatographic separation and enantiomerization kinetics and has become a benchmark example of dynamic chromatography. The detailed mathematical treatment of chromatographic interconversion was established by Eyring, Giddings,^3^ and Keller,^4^ who provided the foundational transport and reaction framework. Later, MacQuarrie introduced the characteristic-function formulation of the stochastic model^5,6^ which greatly simplified numerical treatment and enabled efficient simulation of complex peak behavior. Several comprehensive reviews have discussed this phenomenon and its theoretical and experimental implications in depth.^7–14^

In recent years, several approaches have been proposed to simulate Batman peaks and estimate kinetic and thermodynamic parameters, notably *DCXplorer* ^15^ based on the unified equation model,^16^ *Auto-DHPLC-y2k*,^10^ and the random-walk stochastic methodology of Sepsey et al.^17^ The aim of the present work is therefore not to introduce a new chromatographic theory but to implement established dynamic HPLC models in a unified, reproducible workflow that offers easier application. We have recently shown that both the unified equation model and the stochastic model can be automated in R within a single analysis pipeline.^18^ Without such automation, analysis typically requires laborious manual transfer of data between programs, repeated hand-tuning of fitting, and *ad hoc* postprocessing, all of which reduce reproducibility. By integrating all data analysis processes in one transparent environment, the workflow lowers the practical barrier to applying sophisticated dynamic chromatographic models to routine experimental data sets.

In this work, we extend the previously developed R-based framework to accommodate two-site binding assumptions for chiral stationary phases, enabling a more realistic treatment of enantioselective systems. We further demonstrate how both mobile- and stationary-phase-specific parameters can be estimated directly from HPLC experiments. The resulting workflow is therefore intended as a practical and broadly applicable tool for dynamic chiral separations in which interconversion occurs on the chromatographic time scale.

As a model compound, we selected loratadine, a well-known second-generation antihistamine that exhibits stereochemical behavior not arising from a classical stereogenic center. Instead, loratadine is a conformationally stereolabile tricyclic compound whose nonplanar seven-membered ring gives rise to interconverting enantiomeric conformations. Structurally, the molecule comprises a benzene ring and a pyridine ring fused through a central seven-membered carbocycle and bears an ethylpiperidinecarboxylate substituent. This combination of pronounced conformational mobility and experimentally accessible on-column interconversion makes loratadine an appropriate benchmark for Batman-peak analysis.

Recent work by Mammone et al.^19^ has further clarified the stereochemical stability of loratadine and some of its derivatives, reinforcing the relevance of this scaffold for dynamic chiral studies. At the same time, the present workflow is not restricted to loratadine alone. It could be applicable more generally to other stereolabile systems that display exchange on the chromatographic time scale, including related medium-ring tricyclic scaffolds and other conformationally dynamic pharmaceuticals such as quetiapine^17^ or certain benzodiazepines.^20,21^

## Methods

### Chemicals and High-Performance Liquid Chromatography (HPLC) Measurements

Loratadine as a raw material came from Tokyo Chemical Industry Co., Ltd. (Tokyo, Japan). 1,3,5-Tri*tert*-butylbenzene used as a nonretained marker, with HPLC grade acetonitrile, ethanol, methanol, and diethylamine, were ordered from Merck (Merck KGaA, Darmstadt, Germany).

The chromatograms of loratadine, a commercially available drug that undergoes interconversion, were analyzed. The racemic mixture was injected together with 1,3,5-tri*tert*-butylbenzene as a nonretained reference component in a cellulose column. Chromatographic separations were carried out using an Agilent 1100 chromatographic system consisting of an inline degasser (G1322A), a quaternary pump (G1311A), an automatic injector (G1329A) paired with a sample thermostat (G1330A), a column thermostat (G1316A), and a diode-array detector (G1315A) controlled by Agilent ChemStation B04.03-SP2 software (Agilent Technologies, Waldbronn, Germany). Cellulose-type chiral columns Lux Cellulose-1 (cellulose tris­(3,5-dimethylphenylcarbamate)), Lux Cellulose-2 (cellulose tris­(3-chloro-4-methylphenylcarbamate)), Lux Cellulose-3 (cellulose tris­(4-methylbenzoate)), and Lux Cellulose-4 (cellulose tris­(4-chloro-3-methylphenylcarbamate)) with identical dimensions (150 mm × 4.6 mm; particle size: 5 μm) were the products of Phenomenex (Torrance, CA, USA). The mobile phase consisted of either acetonitrile, ethanol, or methanol containing 0.1% (V/V) diethylamine. Samples for chiral separation were prepared at concentrations of 1.00 mg mL^–1^ loratadine and 0.50 mg mL^–1^ 1,3,5-tri*tert*-butylbenzene in the mobile phase, and 1.0 μL was injected for each run. Loratadine was evaluated on the four cellulose-based chiral stationary phases under a wide range of conditions: column temperature was varied from 10^◦^C to 40^◦^C in 5^◦^C steps, and at each temperature a series of flow rates (0.5, 0.6, 0.7, 0.9, 1.0, and 1.2 mL min^–1^) was applied; for every temperature × flow rate combination, all three diethylamine-modified eluents (acetonitrile, ethanol, and methanol) were tested. For the overload experiments, loratadine solutions were prepared at 5, 10, 20, 50, 100, 200, 300, 400, 500, 1000, 1500, 2000, 5000, 10000, 20000, 50000, and 100000 μg mL^–1^, while the concentration of 1,3,5-tri*tert*-butylbenzene was 0.3 mg mL^–1^. For preparative separation of loratadine, a concentration of 50 mg mL^–1^ was used in methanol. The chromatographic circumstances of preparative separation were as follows: 10 °C, 0.5 mL min^–1^ flow, Lux Cellulose-1 column, and mobile phase methanol containing 0.1% (V/V) diethylamine. UV detection was performed at 225 and 254 nm using a diode-array detector.

### Circular Dichroism (CD) Measurements

CD and UV time-series experiments were performed on a Jasco J-815 spectrometer (Jasco Ltd., Tokyo, Japan) in rectangular quartz Hellma cuvettes with a path length of 1.0 cm.

For the spectrum measurements, the slit was set to 2 nm, the registration speed to 100 nm min^–1^, and the accumulation to 3. Blank correction was applied with the solvent (acetonitrile, ethanol, or methanol containing 0.1% (V/V) diethylamine). The first fraction of preparative separation runs was collected for 24 ± 2s (the start of which marked *t*_0_ of the racemization kinetics), which was diluted to obtain an absorbance of approximately 1.5. The ellipticity data were read at a circular dichroism peak maximum of 248.5 nm. During the time course measurement, a measurement point was acquired every 2 s using a digital integration time of 500 ms. Temperature control was achieved by using a Jasco CDF-426L cuvette thermostat. For the measurements, the cuvette was thermostated to 5, 10, 15, 20, 25, and 30 °C and the actual temperature of the solution was measured and used for the calculations.

### Computational Chemistry Methods

All computations were carried out with the Schrödinger 2025–3 program package.^22^ Structures were drawn with Schrödinger’s Maestro graphical user interface (GUI).^23^ Initial minimizations and conformational searches were completed with MacroModel using the OPLS4 force field, with no solvent.^24^ The Mixed torsional/Large scale low-mode sampling with standard settings was used for conformer generation. The two lowest energy conformations pertain to the enantiomers: conformer “a” with a −53.4° dihedral in the piperidine ring and a −72.0° dihedral in the cycloheptane ring; conformer “b” with a 53.4° dihedral in the piperidine ring and a 72.0° dihedral in the cycloheptane ring.

Geometry optimizations were carried out for the selected conformers using Schrödinger’s quantum mechanics (QM) engine, Jaguar.^25^ The density functional theory (DFT) B3LYP-D3 method was used with the 6-31G** basis set; default settings were used. Both conformers were optimized with methanol, ethanol, and acetonitrile polarizable continuum model (PCM) as the solvation protocol.^26^

Based on the optimized conformer structures, transition state searches were completed with Jaguar’s linear synchronous transit (LST) method with the LACVP basis set and ** polarization in all three solvents. With this method, the transition state is searched as a maximum on the potential energy surface along a linear path connecting conformer “a” as “reactant” and conformer “b” as “product” structures.

Jaguar-relaxed coordinate scan was used to perform constrained geometry optimizations based on dynamically adjusted coordinates with the (DFT) B3LYP-D3 method and 6-31G** basis set. The above-mentioned dihedrals were varied between −53.4° to 53.4°, and −72.0° to 72.0° in nine steps each; in total, a series of 91 geometries were generated and optimized inthe methanol PMC solvent model, and then the whole process was repeated with ethanol and acetonitrile solvent models .

### Data Analysis and Code Script

All analyses were performed using custom scripts written in R version 4.5.1 ^27^ and developed in RStudio Version 2025.09.0 + 387 (Posit, Boston, MA, USA). The full code is openly available on GitHub: https://github.com/mirzahosseini-arash-semmelweis/Batman_chromat. The complete, fully commented code is also provided in the Supporting Information, along with the experimental data, so that key results can be reproduced directly.

The foundational elements of the Batman algorithm, together with the underlying libraries employed,^28–31^ were described in our previous work.^18^ Here, we present an improved and substantially augmented implementation, incorporating enhanced numerical stability, extended model capabilities, and additional optimization constraints tailored to stochastic peak-shape analysis. In brief, the algorithm (after reading the data: time vs detector intensity) identifies the nonretained component peak (if present) and the characteristic Batman peak (peak A and peak B, the first and second eluted species). Chromatographic parameters are then estimated (number of theoretical plates *N*, retention time *t*_*i*_, full width at half-maximum *w*_*i*_, peak height *h*_*i*_, plateau relative height *h*_p_, and peak areas *A*_∞_ and *B*_∞_), which in turn afford the kinetic constant from the unified eq 1 as follows:

1 $$k_{\mathrm{ue},f}=-\frac{1}{t_{A}}[\log\left(\frac{100\left(1-A_{0}\right)+A_{0}\left(100-h_{p}\left(1+\sqrt{\frac{2}{\pi N}}\right)\right)}{t_{B}-t_{A}}\right)-\log(\left(1-A_{0}\right)\left(\frac{h_{p}e^{-{\left(t_{B}-t_{A}\right)}^{2}/2s_{B}^{2}}-100e^{-{\left(t_{B}-t_{A}\right)}^{2}/8s_{B}^{2}}}{s_{B}\sqrt{2\pi }}+\frac{100}{t_{B}-t_{A}}\right)-A_{0}\left(\frac{100e^{-{\left(t_{B}-t_{A}\right)}^{2}/8s_{A}^{2}}-h_{p}}{s_{A}\sqrt{2\pi }}+\frac{h_{p}\left(1+\sqrt{\frac{2}{\pi N}}\right)-100}{t_{B}-t_{A}}\right))]$$

*A*_0_ is the fraction of enantiomer A in the injected sample and is provided *a priori*. The rate constant for the reverse direction is calculated as follows using eq 2:

2 $$k_{\mathrm{ue},r}=k_{\mathrm{ue},f}\frac{A_{\infty }}{B_{\infty }}\frac{t_{A}}{t_{B}}$$

The characteristic function approach to the stochastic theory of chromatography is next applied to model the separation process as a composite Poisson process with exponentially distributed sojourn (residence) times. The characteristic function, as the Fourier transform of the elution profile, contains all necessary information about the peaks expressed as eq 3:

3 $$\Phi \left(\omega \right)=\exp\left(n\left(\frac{1}{1-i\tau \omega }-1\right)\right)$$

where *n* is the expected number of adsorption–desorption steps, τ is the expected time spent by a molecule bound to the stationary phase (mean sojourn time), and ω is an auxiliary real variable (frequency). The peak profile of the Batman peaks can be obtained by the convolution of the elution curves resulting from the characteristic function with a weighted sum of conditional probability densities (eq 4) given by the Keller–Giddings distribution as follows:

4 $$\begin{array}{r} P_{\mathrm{AA}}\left(x\right)=\sqrt{\frac{ab\left(1-x\right)}{x}}\exp\left(-a\left(1-x\right)-bx\right)I_{1}\left(\sqrt{4abx\left(1-x\right)}\right)\\ P_{\mathrm{AB}}\left(x\right)=a\exp\left(-a\left(1-x\right)-bx\right)I_{0}\left(\sqrt{4abx\left(1-x\right)}\right)\\ P_{\mathrm{AA}}^{\prime }=\delta_{A}\exp\left(-a\right) \end{array}$$

where *x* is the fraction of time that a molecule spends as A, *I*_*i*_ is the modified Bessel function of the first kind and *i*th order, δ_*i*_ is the Dirac delta, and *a* and *b* are the expected number of interconversions from A to B and vice versa. The above probabilities correspond to the 3 possible states of a species (beginning in state A) at the end of the run: being in the same state with no interconversion having occurred; being in the same state with at least two interconversion events having occurred; and being in the opposite state (state B). The same distributions can be defined for starting state B by exchanging the roles of *a* with *b*, and *x* with 1 – *x*.

For fitting the Batman peaks with the stochastic model, the chromatograms were first deconvoluted from the peak of the nonretained marker (modeled with an exponentially modified Gaussian distribution). The fitting proceeded in two stages: first, a differential evolutionary genetic algorithm was applied to refine the parameters toward the optimal region; this was followed by a Levenberg–Marquardt optimization with a warm start to achieve tighter convergence. To improve numerical robustness, several updates were implemented in the fitting workflow. Linear regression steps used in batch evaluation were rewritten using a safeguarded formulation to prevent numerical instabilities or failures. A final normalization step was introduced in the Batman peak simulation functions to ensure consistent scaling between the observed and simulated chromatograms. Preprocessing routines were extended to include adaptive interpolation and extrapolation of chromatographic parameters, together with improved initial estimation of the stochastic mixing parameters *a* and *b*. Finally, explicit box constraints were imposed on the stochastic model parameters during both global and local optimization stages, ensuring physically meaningful solutions and preventing *a* and *b* from drifting outside the admissible ranges during Levenberg–Marquardt refinement. These improvements increased the convergence success rate to 100%.

## Results

### HPLC Experiments: Separation under Different Conditions and Overload Studies

Loratadine was chromatographically evaluated on four coated cellulose-based chiral stationary phases across a wide range of experimental conditions described in the Methods section. This systematic design provided a comprehensive data set to evaluate the effects of thermal activation, mass-transfer modulation, and solvent–selector interactions on the enantiomerization behavior of loratadine. Clear trends were observed across the entire experimental matrix. As expected based on prior observations, the extent of on-column interconversion increased markedly with temperature, leading to progressive coalescence of the enantiomeric peaks. Flow rate variation produced the anticipated kinetic modulation: faster flow rates reduced the residence time on the chiral stationary phase, thereby suppressing the degree of interconversion and yielding more distinct peaks.

More pronounced differences emerged between eluents. With alcohol-type eluents, enantioseparation was achieved on all investigated columns, whereas in acetonitrile, separation was observed only on Lux Cellulose-2 and Lux Cellulose-4. On Lux Cellulose-1 and Lux Cellulose-3, no enantiorecognition was detected in acetonitrile under any condition. Acetonitrile nevertheless consistently produced the fastest apparent interconversion kinetics, often approaching near-instantaneous equilibration in some phases. Notably, this acceleration occurred despite acetonitrile generally exhibiting higher retention, in contrast to the ethanol–methanol comparison, where faster interconversion tended to coincide with lower retention. Although a direct correlation between retention and interconversion cannot be generalized, this contrasting behavior points to distinct solvation and interaction mechanisms in acetonitrile compared with alcohol-type eluents. In contrast, alcohol eluents exhibited slower kinetics overall across the entire temperature and flow-rate range. Representative chromatograms illustrating these behaviors are shown in Figure 1 (left panel).

**Figure 1:**
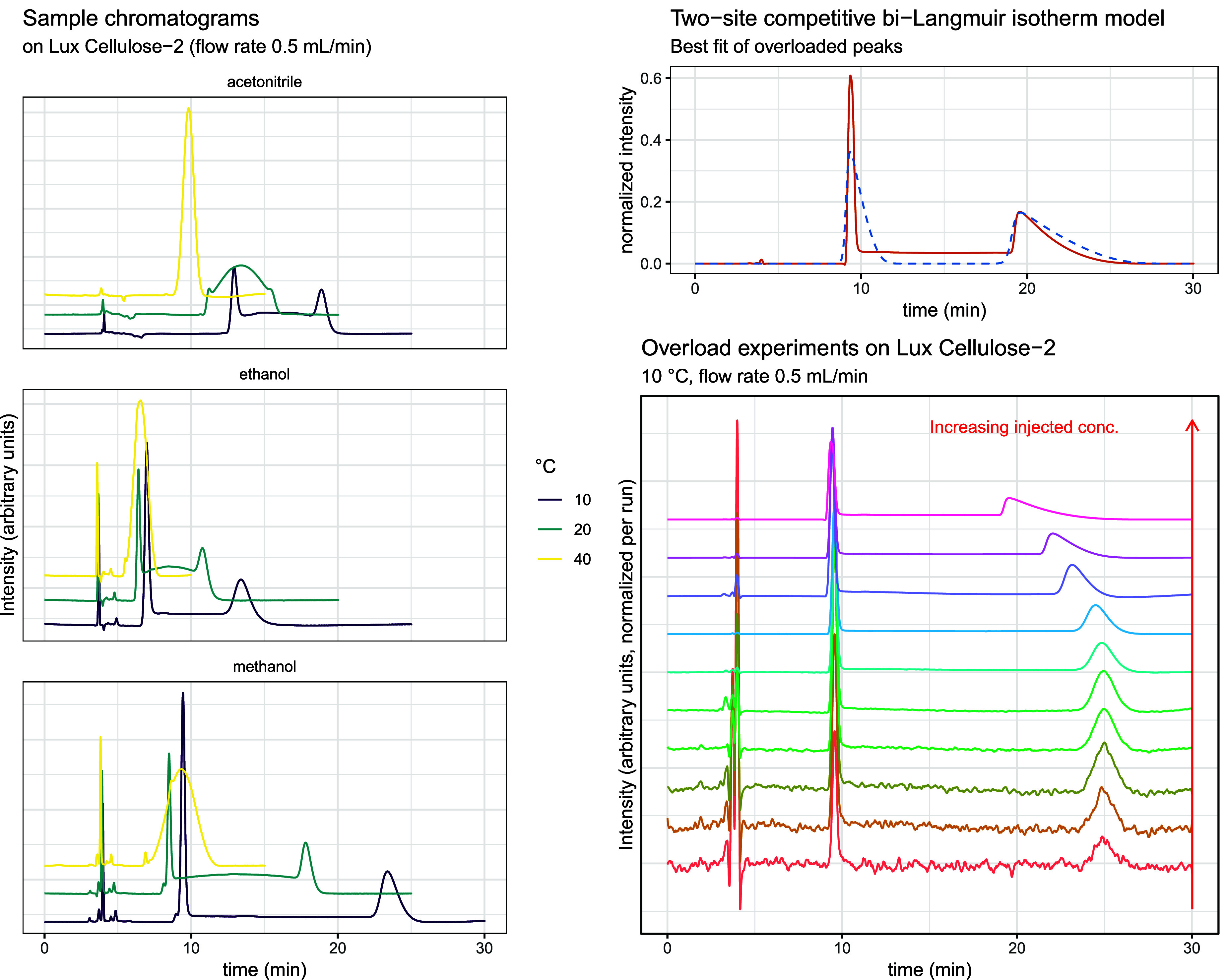
Left: Example chromatograms were obtained on a cellulose-based chiral stationary phase at selected temperatures. The traces illustrate the pronounced dependence of on-column enantiomerization on the temperature and the strong eluent-specific differences. Right: Representative overload peak profiles were recorded in methanol on the same column, together with the best fit of the two-component competitive bi-Langmuir isotherm model.

To further characterize sorption behavior and to extract adsorption-isotherm parameters, a series of overload experiments was performed. These were conducted exclusively in methanol at 0.5 mL min^–1^ across all studied temperatures and columns, using injected loratadine concentrations spanning from 0.005 mg mL^–1^ to 100 mg mL^–1^. Representative overload peak profiles are presented in Figure 1 (right panel).

### Isotherm Analysis

The overloaded chromatograms of the racemic mixture were analyzed using a competitive bi-Langmuir isotherm embedded in an equilibrium-dispersive column model.^32,33^ Two classes of adsorption sites were considered: a nonselective site (ns), characterized by identical affinity toward both enantiomers, and a selective site (s), exhibiting different affinities. The equilibrium loadings were described by eq 5 as follows:

5 $$q_{i}=Q_{\mathrm{ns}}\frac{b_{\mathrm{ns}}c_{i}}{1+b_{\mathrm{ns}}\left(c_{1}+c_{2}\right)}+Q_{s}\frac{b_{s,i}c_{i}}{1+b_{s,1}c_{1}+b_{s,2}c_{2}},,i=1,2$$

where *Q*_ns_ and *Q*_s_ denote the respective site capacities. and *b*_ns_, *b*_s,1_, and *b*_s,2_ the affinity coefficients. The capacities were reparameterized as *Q*_tot_ = *Q*_ns_ + *Q*_s_ and ρ = *Q*_s_/*Q*_ns_, enabling direct estimation of the site ratio ρ.

Although this formulation assumes identical selective-site capacities for both enantiomers, deviations from this assumption may occur in practice. In such cases, the model can be generalized by introducing separate selective capacities for each enantiomer. However, this extension increases the number of adjustable parameters and typically leads to strong parameter correlation and reduced identifiability. This variant with independent selective capacities was also evaluated, but it did not lead to a meaningful improvement in the quality of the fits.

Chromatographic transport of the overloaded peaks was described by the equilibrium-dispersive model (eq 6),

6 $$\varepsilon \frac{\partial c_{i}}{\partial t}+\left(1-\varepsilon \right)\frac{\partial q_{i}}{\partial t}+u\frac{\partial c_{i}}{\partial z}=D_{\mathrm{ax}}\frac{\partial^{2}c_{i}}{\partial z^{2}}$$

where *ε* is the void fraction/porosity, *u* is the interstitial velocity, *t* is the time, *z* is the axial position, *c* is the mobile phase concentration, *q* is the adsorbed amount, and *D*_ax_ the axial dispersion coefficient. The latter was linked to column efficiency via $D_{\mathrm{ax}}=\frac{uL}{2N}$ with *L* denoting column length and *N* the number of theoretical plates. The equilibrium-dispersive model coupled with the competitive bi-Langmuir process was numerically integrated on a discretized spatial domain with Danckwerts inlet and zero-gradient outlet boundary conditions. Details of the algorithm are discussed in detail^34^ and applied recently.^35^

Global fitting yielded a consistent parameter set across the injections with peaks exhibiting column overload, with a representative fit depicted in Figure 1 (right panel). The simulations adequately reproduced (i) the pronounced tailing of the first-eluted enantiomer, (ii) the relative position of the second-eluted peak, and (iii) the relative signal magnitudes. Not all chromatograms exhibited clear overload, and the agreement was not perfect across all injection levels. Nevertheless, the model provided robust estimates of the relative site capacities. The resulting site ratio (ρ: 15–30) was in good agreement with literature values reported for comparable chiral stationary phases^36^ and was used to define physically meaningful bounds for stochastic parameter estimation.

### Phase-Specific Kinetic Constants Decomposition by Mixed-Effects Modeling

Following the extraction of site capacity ratios from the overload experiments, the Batman fitting with the stochastic model was repeated using a two-site characteristic function to account explicitly for nonselective and enantioselective sites. In the original one-site formulation,^18^ the number of interconversion events is described by a single exponential term in the characteristic function. In the two-site case (eq 7), this was generalized to

7 $$\Phi \left(\omega \right)=\exp\left(n\left(\frac{p_{1}}{1-i\tau_{1}\omega }+\frac{p_{2}}{1-i\tau_{2}\omega }-1\right)\right)$$

where *p*_1_ and *p*_2_ are the abundance ratios of nonselective and enantioselective sites, respectively, and τ_1_ and τ_2_ are the concomitant mean sojourn times. In Figure 2, representative fits of the one- and two-site stochastic models are compared. The two-site model consistently produces a tighter fit to the experimental peak shape, which is expected given its additional degrees of freedom. This increase in flexibility was reflected in an improvement in peak shape recognition. However, in a minority of cases additional flexibility also led to overfitting, manifested as implausible parameter estimates with high correlation, or unusually large uncertainties. These fits were flagged and handled during the subsequent pooling and outlier-detection steps. Importantly, the incidence of overfitting could be drastically reduced by imposing explicit box constraints during the Levenberg–Marquardt refinement stage and by initializing the optimization with physically sensible parameter values extrapolated from reliable peak shapes recorded under conditions of low interconversion.

**Figure 2:**
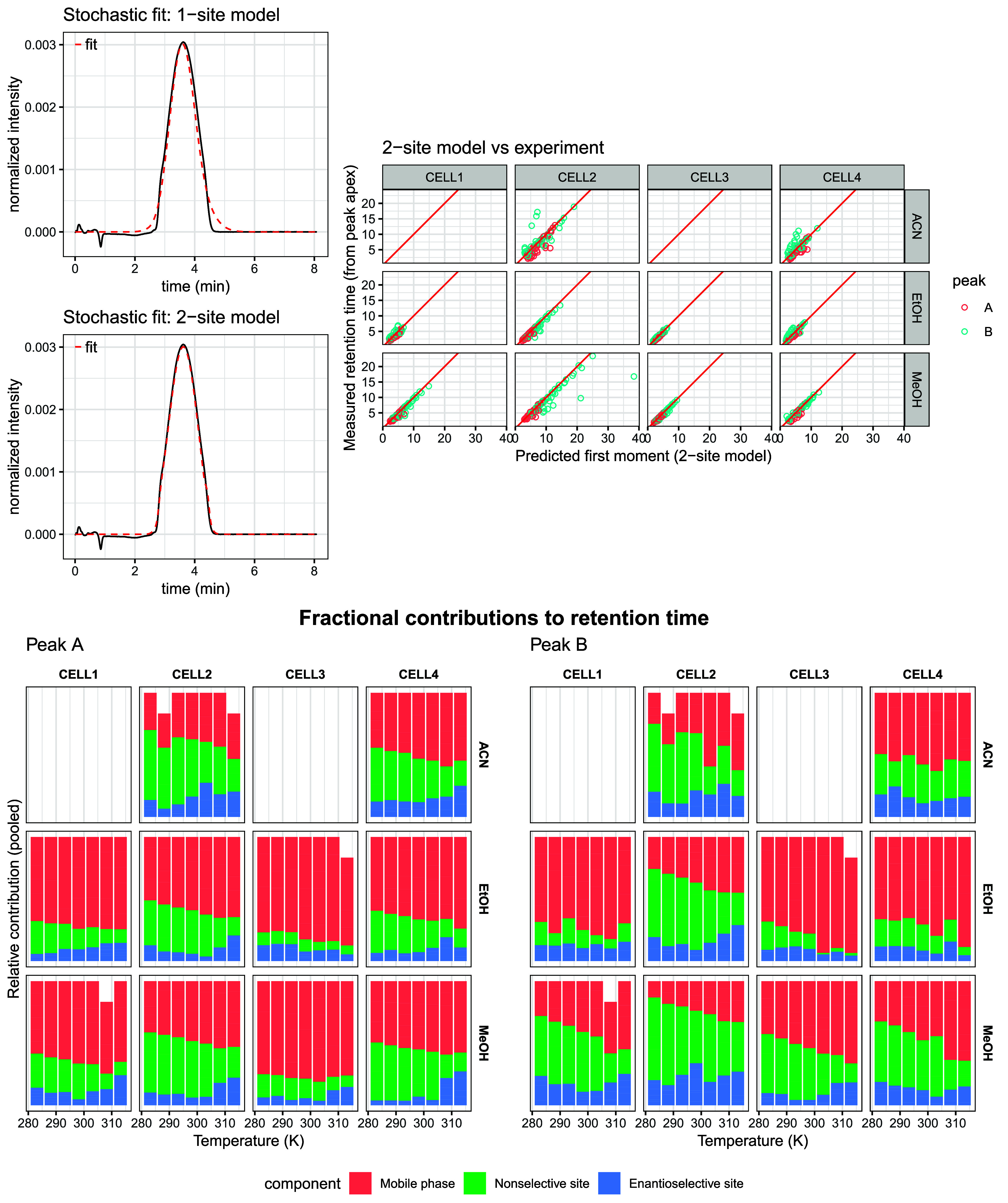
Top left: Comparison of 1-site and 2-site stochastic model fits with conditions: Lux Cellulose-2, acetonitrile, 1 mL/min, 30 °C. Top right: Comparison of total retention time from 2-site model parameters vs peak apex time. Lines of equality are shown in red. Bottom: Contributions of mobile phase and 2-site stationary phase retention across temperature and eluent conditions for the two peaks (pooled across all flow rates and stationary phases).

Apart from these occasional overfitting artifacts, the two-site framework remained physically meaningful. The theoretical retention times predicted from the fitted two-site parameters were in very good agreement with the experimentally observed peak apex positions across all temperatures, columns, and eluents (Figure 3). This agreement supports the internal consistency of the two-site parametrization even in cases where individual stochastic parameters were weakly determined. A stacked decomposition of the phase- and site-specific contributions to the total residence time provided further mechanistic insight. Across all phases, the second-eluting peak exhibited a larger fractional residence time on the enantioselective site, whereas the first-eluting peak sampled this site only weakly.

**Figure 3:**
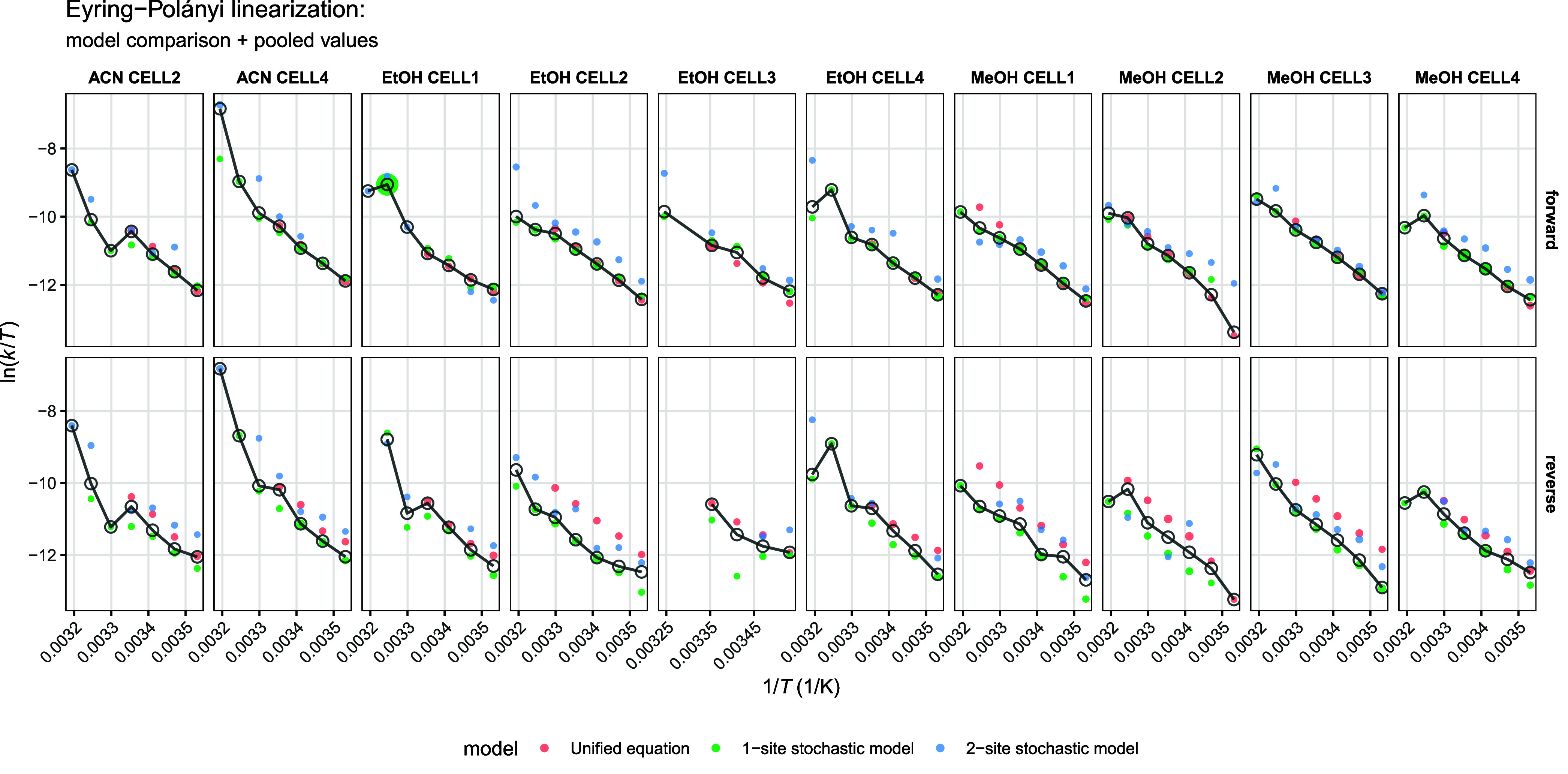
Apparent kinetic constants obtained from each model and their pooled estimates are shown in Eyring–Polányi linearized form across all chromatographic conditions. The size of each model estimate point is proportional to its standard error.

Fitting of the chromatograms was also performed with a one-site stochastic model, as well as the unified equation (UE). For each chromatogram, forward and reverse interconversion rate constants were obtained from each model and then pooled across the experiments. Outlier detection was implemented at the level of the grouped data, but no statistically problematic points were identified. The pooled UE rate constants *k*_ue_,*f* and *k*_ue_,*r* were summarized by group means and standard errors, and analogously for the reverse direction, providing a first, model-specific estimate of the interconversion kinetics.

Stochastic kinetic parameters were then extracted from the coefficients of the characteristic function. In the one-site stochastic model, the forward and reverse rate constants were determined from simple linear regressions (after outlier detection) of the form *a* = *k*_stoch,*f*_ *t*_A_ and *b* = *k*_stoch,*r*_ *t*_B_, where *t*_A_ and *t*_B_ are the enantiomer-specific retention times. This reflects the assumption that in the one-site approximation interconversion is effectively averaged over the total residence time of each enantiomer in the column. For the two-site stochastic model, the regression was extended to $a=k_{\mathrm{stoch},f}^{\left(2\right)}\left(t_{M}+p_{2}n_{A}\tau_{A2}\right)$ and $b=k_{\mathrm{stoch},r}^{\left(2\right)}\left(t_{M}+p_{2}n_{B}\tau_{B2}\right)$ , where *t*_M_ is the void or holdup time. This formulation explicitly encodes the mechanistic assumption that interconversion occurs predominantly in the mobile phase and on the enantioselective sites rather than uniformly across all accessible phases.

To obtain robust kinetic constants, the rate estimates from the above three methods were combined by using inverse-variance weighting. Across all experimental conditions, the across-model consistency of the kinetic constants was high. Model-level outliers were quantified by comparing the values from each model to the within-group median. Diagnostic plots with Eyring–Polányi linearization (Figure 3) show that the pooled *k* values lie centrally within the spread of individual model estimates, with no systematic bias toward any single modeling framework. This indicates that the unified equation and both stochastic implementations are mutually compatible in their kinetic interpretation.

To decompose the pooled apparent rate constants into phase-specific kinetic contributions, we next used the theoretical relationship that the observed forward and reverse interconversion rates are capacity factor (*k*_fact_) weighted mixtures of mobile phase and stationary phase rate constants. Because the observed apparent interconversion rate *k*_obs_ depends on the residence distribution between the mobile phase and stationary phase, we follow the standard decomposition as expressed in eq 8:

8 $$k_{\mathrm{obs}}=\frac{1}{1+k_{\mathrm{fact}}}k_{\mathrm{mob}}+\frac{k_{\mathrm{fact}}}{1+k_{\mathrm{fact}}}k_{\mathrm{stat}}$$

where *k*_mob_ and *k*_stat_ denote the mobile phase and stationary phase interconversion rate constants, respectively. It is further assumed that *k*_mob_ is identical in both interconversion directions, whereas *k*_stat_ is direction-dependent and corresponds to either *k*_stat,forward_ with *k*_fact,A_ or *k*_stat,reverse_ with *k*_fact,B_ in the equation above. Finally, the model assumes that mobile phase and stationary phase interconversion kinetics are independent and noninteracting. Using the pooled rate constants *k*_pool_ with their uncertainties, we defined a transformed response *k*_tr_ = *k*_pool_ (1 + *k*_fact_) that linearizes the relationship for mixed-effects modeling and permits direct estimation of *k*_mob_ and *k*_stat,forward/reverse_. The transformed rates were modeled using a weighted linear mixed-effects model^37^ in the form of eq 9:

9 $$k_{\mathrm{tr},ijkd}=\beta_{0}+\beta_{f}k_{\mathrm{fact},ijk}+\beta_{\Delta }k_{\mathrm{fact},ijk}1_{\{d=\mathrm{reverse}\}}+u_{i,j}+v_{k,j}k_{\mathrm{fact},ijk}+\varepsilon_{ijk}$$

where indices denote the following:

- *i*: eluent,
- *j*: temperature,
- *k*: column,
- *d* ∈ {forward, reverse} indicates interconversion direction.

Fixed effects:

- β_0_: global intercept—mobile phase rate (assumed to be the same in both directions)
- β_*f*_: global slope for forward direction—stationary phase forward rate
- β_Δ_: difference between forward and reverse slopes—adds to β_*f*_ to give the reverse stationary phase rate
- **1**_{*d* = reverse}_: indicator function equal to 1 when the observation corresponds to the reverse direction and 0 otherwise.

Random effects:

- *u*_*i,j*_: random intercept for each eluent–temperature combination
- *v*_*k,j*_: random slope adjustment for each column–temperature pair
- *ε*_*ijk*_: residual error

This mixed-effects model describes how the observed interconversion rate changes with stationary-phase contribution and direction of conversion while also allowing for systematic variation between eluents, temperatures, and columns. This allows decomposition of apparent rate constants into the underlying phase-specific parameters.

The error term ε_*ijk*_ was modeled with heteroskedastic weights based on the capacity factor. This variance structure accounts for the increased uncertainty when the stationary phase contribution becomes appreciably weighted, consistent with the propagation of uncertainty (calculated using arm::se.ranef^38^) from the pooled rate constants. Mobile phase rate constants extracted from the fixed intercept exhibited well-behaved Eyring–Polányi plots. In contrast, stationary phase rate constants were smaller in magnitude, leading to high noise despite the successful convergence of the mixed-effects model. Consequently, stationary phase-specific parameters were most reliably determined when decomposition was restricted to the unified-equation rate constants, which showed the lowest intrinsic variance. This approach produced consistent stationary phase estimates while retaining the strong interpretability of the mixed-effects decomposition. The decomposed phase-specific kinetic constants are shown with Eyring–Polányi plots in Figure 4, while the diagnostics of the mixed-effects model are depicted in Figure 5. The complete list of fitted phase-specific rate constants is available in the Supporting Information.

**Figure 4:**
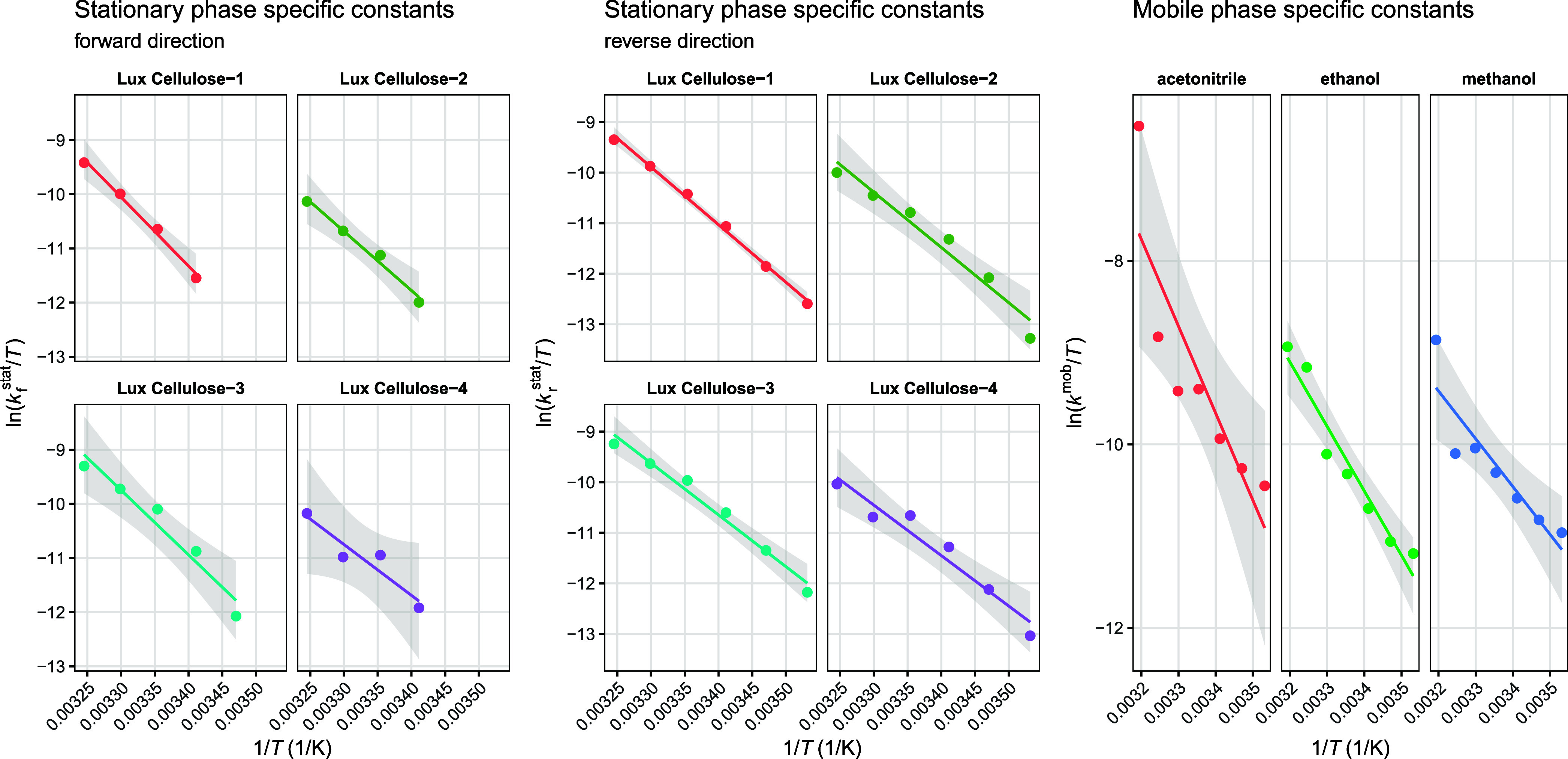
Eyring–Polányi linearized plots (with confidence bands) of the phase-specific rate constants.

**Figure 5:**
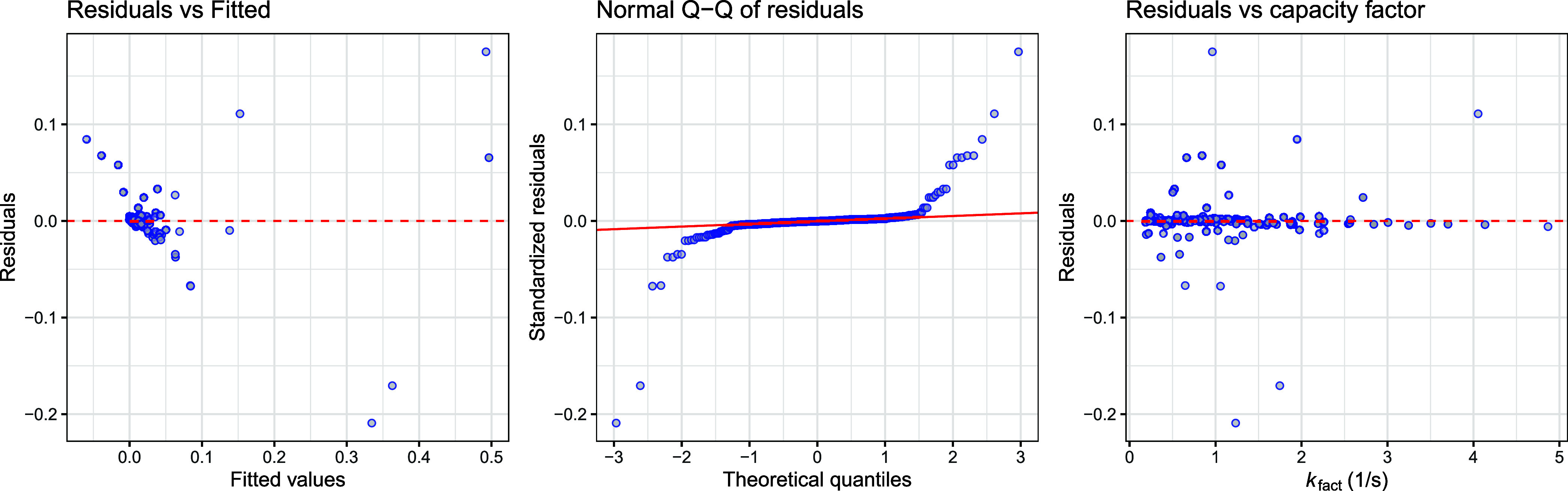
Diagnostic plots of the mixed-effects model. Some variance dependence of residuals is still observed, but the model did not benefit from more sophisticated variance structures.

### Thermodynamic Parameters from Mixed-Effects Model

Activation thermodynamics (enthalpy Δ*H* ^‡^ and entropy Δ*S* ^‡^) for both forward and reverse interconversion were obtained by applying the Eyring–Polányi equation in its linearized form (eq 10):

10 $$\ln\left(\frac{k}{T}\right)=-\frac{\Delta H^{‡}}{R}\frac{1}{T}+\ln\left(\frac{k_{B}}{h}\right)+\frac{\Delta S^{‡}}{R}$$

so that weighted linear regression yields the slope *m* = −Δ*H* ^‡^/*R* and intercept *b* = ln­(*k*_B_/*h*) + Δ*S* ^‡^/*R*, where *R*, *h*, and *k*_B_ are the universal gas, Planck, and Boltzmann constants, and *T* is the absolute temperature. Because each kinetic constant is associated with an uncertainty, the regression of thermodynamic parameters propagated this uncertainty using first-order error propagation and weighted linear regression so that points with a smaller relative error contribute proportionally more to the fit. The apparent and phase-specific activation enthalpies and entropies (reported in kJ mol^–1^ and kJ K^–1^ mol^–1^) are depicted in Table 1. All fitted thermodynamic parameters (apparent and phase-specific together with uncertainties) are included in the Supporting Information.

**Table 1:** Activation Enthalpies (Left Panel) and Entropies (Right Panel) Reported in kJ mol^–1^ and kJ K^–1^ mol^–1^ Obtained from Weighted Eyring–Polányi Analysis for the Forward (Top Panel) and Reverse (Bottom Panel) Interconversions^a^

		Enthalpy of activation					Entropy of activation				
Direction		Cell-1	Cell-2	Cell-3	Cell-4	Mob. phase	Cell-1	Cell-2	Cell-3	Cell-4	Mob. phase
Forward	Acetonitrile	NA	82.1	NA	81.9	161.9	NA	–0.01	NA	–0.01	0.27
	Ethanol	91.9	60.4	65.3	68.9	65.6	0.03	–0.09	–0.07	–0.06	–0.06
	Methanol	65.2	82.1	66.4	61.3	69.1	–0.07	–0.01	–0.06	–0.08	–0.05
	Stat. phase	95.5	81.9	70.1	71.4		0.03	–0.02	–0.05	–0.05	
Reverse	Acetonitrile	NA	59.9	NA	95.9	161.9	NA	–0.09	NA	0.04	0.27
	Ethanol	79.8	67.2	67.9	84.6	65.6	–0.02	–0.07	–0.06	–0.00	–0.06
	Methanol	63.3	76.1	73.1	57.9	69.1	–0.08	–0.04	–0.05	–0.10	–0.05
	Stat. phase	85.1	66.6	65.9	59.9		0.00	–0.06	–0.06	–0.09	

a Apparent activation enthalpies and entropies are listed in the margin of each panel, whereas the phase-specific values are shown within the body. Standard error quartiles: apparent enthalpies 1.5–4.75; apparent entropies 0.005–0.02; phase-specific enthalpies 4–9; phase-specific entropies 0.01–0.03.

### Validation with Circular Dichroism Measurements

To validate the kinetic parameters obtained from HPLC, independent CD measurements were performed in the same mobile phases by using isolated fractions of the first-eluting enantiomer. For each temperature, the collected fraction was rapidly transferred to the cuvette under temperature control and the decay of the CD signal (together with the corresponding UV absorbance) was recorded as a function of time. Since the start of data acquisition did not exactly coincide with the moment of dissolution, the kinetic model was written in terms of the true reaction time. In addition, small calibration offsets between the nominal and actual temperatures were corrected. At each corrected temperature, the CD signal was modeled as a simple first-order decay as in eq 11:

11 $$\theta \left(t\right)=\theta_{0}e^{-k_{\mathrm{mob}}t}$$

and the parameters θ_0_ (ellipticity of the enantiomer) and *k*_mob_ were estimated by nonlinear least-squares. For visualization and quality control, the time-series fits in methanol at all temperatures are shown in Figure 6 (left), together with the experimental CD traces.

**Figure 6:**
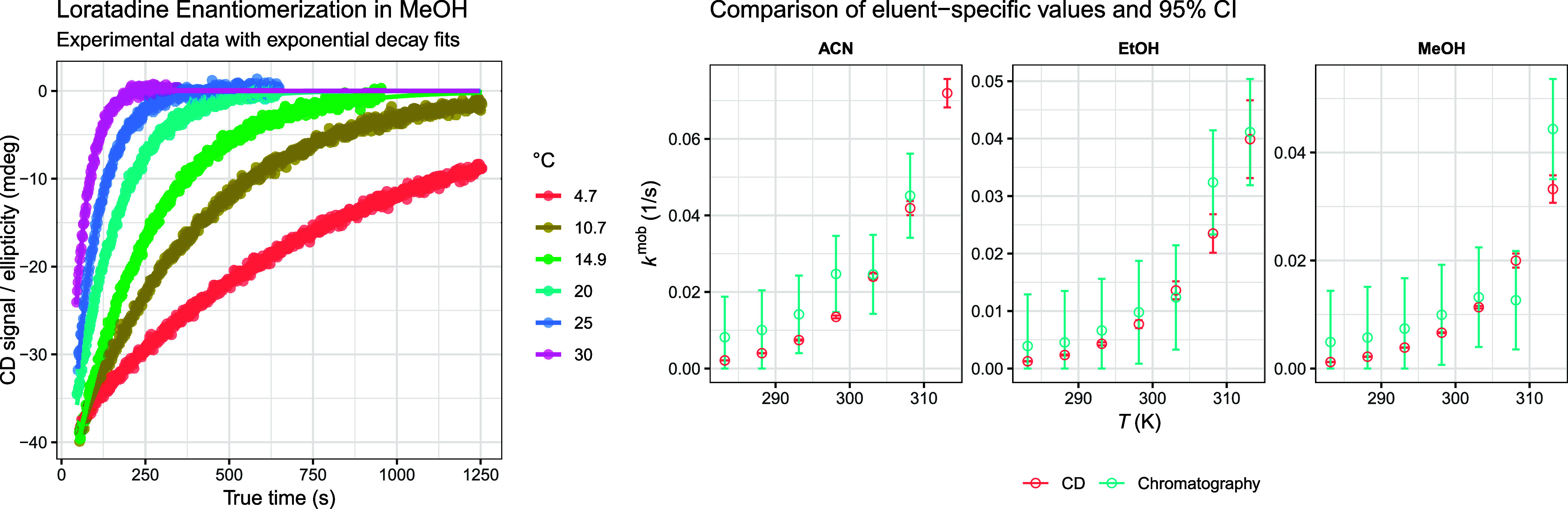
Left: Circular dichroism time-series measurements for loratadine in methanol at the indicated temperatures were together with first-order exponential decay fits. The time axis represents the true kinetic time, corrected for temperature-dependent delays between fraction collection and the start of acquisition. Right: Comparison of mobile phase-specific interconversion rate constants obtained independently from HPLC analysis (mixed-effects decomposition) and CD kinetics. Statistical equivalence between the two methods is assumed when the corresponding confidence intervals overlap. For visual clarity only, the confidence intervals associated with the mixed-effects decomposition estimates are displayed after uniform downscaling by a factor of 5; this graphical rescaling does not affect either the underlying statistical comparison or the conclusions drawn from the data.

The temperature dependence of the CD-derived rate constants was then analyzed using an Arrhenius-type relationship (eq 12):

12 $$\ln k_{\mathrm{mob}}=\ln A-\frac{E_{a}}{R}\frac{1}{T}$$

where *A* is the Arrhenius factor and *E*_a_ the apparent activation energy. A weighted linear regression of ln *k* versus 1/*T* afforded the mobile phase-specific rate constants. Finally, the CD-based rate constants were scaled by a factor of $\frac{1}{2}$ to convert from the overall racemization rate to the unidirectional mobile phase enantiomerization rate, consistent with the kinetic convention used in the HPLC analysis. In parallel, the ratio of the extrapolated CD amplitude θ_0_ to the long-time absorbance was used to estimate the *G*-factor with uncertainty obtained by standard error propagation (provided in the Supporting Information). These CD-derived mobile phase rate constants and their uncertainties were then directly compared to the mobile phase-specific rate constants extracted from HPLC (mixed-effects decomposition) at matching temperatures. Figure 6 (right) shows this comparison, demonstrating good agreement between the independently determined CD and chromatographic kinetic parameters over the full temperature range.

### Empirical Bayesian Inference

To further refine the fitting of phase-specific kinetic parameters across the full experimental space simultaneously, an empirical Bayesian (EB) framework was applied using mixed-effect modeling results as informative priors. In contrast to the run-by-run fitting approaches described above, this strategy treats all chromatograms jointly and estimates phase-specific interconversion kinetics in a unified model, while explicitly accounting for temperature dependence and experimental heterogeneity. For each chromatographic run, experimentally determined peak moments and normalized intensity–time profiles were used as inputs. The underlying peak profiles were approximated by Gaussian distributions, and the two peak profiles were convoluted with Giddings interconversion probability distributions. Interconversion kinetics were described by forward and reverse stationary phase rate constants together with an eluent-dependent mobile phase contribution. The temperature dependence of all rate constants was parametrized using a linearized Eyring–Polányi formulation referenced to a common temperature (the median of the studied temperature range), allowing information to be shared across runs recorded at different temperatures and flow rates.

Global parameter estimation was performed by maximizing the joint posterior density (*maximum a posteriori* estimation). This empirical Bayesian approach effectively pools information across the entire data set, shrinking poorly informed parameters toward physically reasonable values, while allowing well-informed parameters to be driven by the data. Convergence was assessed by monitoring the objective function (sum of squared error terms across all runs) and by verifying the numerical stability of the fitted Hessian matrix. From the fitted global parameters, run-specific interconversion counts were reconstructed and used to generate predicted chromatographic profiles (Figure 7). Diagnostic plots showed no systematic dependence of residuals.

**Figure 7:**
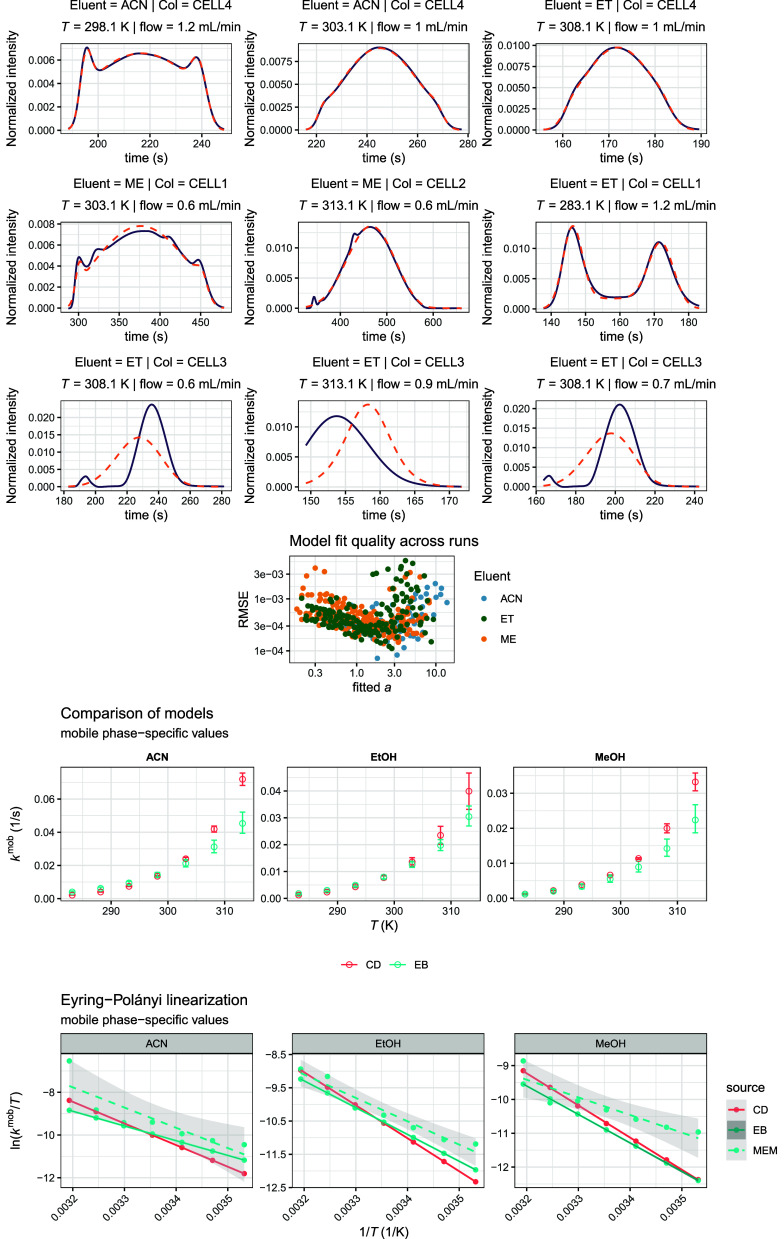
Empirical Bayesian (EB) model performance. Top: Representative fits (best/median/worst SSE by rows). Center: Root-mean-square error (RMSE) versus the fitted interconversion count parameter, illustrating the model fit quality across all experiments. Bottom: Direct comparison of the kinetic constants shows agreement between CD-derived values and EB estimates. Eyring–Polányi linearization reveals improved consistency of CD data with EB-derived eluent-specific kinetics relative to those obtained from the mixed-effects model (MEM). Confidence bands for CD and EB (the latter are Hessian-based propagation) are also shown but are narrow at this scale.

Uncertainty quantification was carried out using a Laplace (curvature-based) approximation derived from the Hessian at the optimum (fitted parameters and analysis script are compiled in the Supporting Information). Column-specific forward and reverse activation enthalpies and entropies were obtained directly from the fitted temperature slopes and intercepts, while eluent-specific activation parameters characterized the mobile-phase-mediated contribution to interconversion (compiled in Table 2). Standard errors reflected both data variability and parameter correlations inherent to the global model. Comparison with the alternative analysis strategy of a mixed-effects model (MEM) highlighted the advantages of the EB framework. While run-by-run frequentist fits followed by secondary mixed-effect modeling yielded qualitatively similar trends, they exhibited larger dispersion and occasional nonphysical estimates, particularly for runs with weakly identifiable kinetics (Figure 7, bottom row). In contrast, empirical Bayesian estimates were more stable across the experimental domain and closely matched results obtained from fully Bayesian hierarchical modeling (results not shown, but Stan code provided in Supporting Information), while avoiding the computational burden and surrogate-function instability encountered in the latter.

**Table 2:** Activation Enthalpies, Entropies, and Gibbs Energies at 298.15 K (in kJ mol^–1^, kJ K^–1^ mol^–1^, and kJ mol^–1^, Respectively) Obtained from Empirical Bayesian Inference for the Forward and Reverse Interconversions in Stationary Phase and Mobile Phase^a^

Phase	Δ*H* ^‡^	Δ*S* ^‡^	Δ*G* ^‡^
**Stat. Forward**			
Cell-1	78.3	–0.02	85.6
Cell-2	79.6	–0.03	88.0
Cell-3	70.9	–0.05	84.3
Cell-4	76.5	–0.03	86.7
**Stat. Reverse**			
Cell-1	90.3	0.01	88.3
Cell-2	85.2	–0.02	90.2
Cell-3	73.7	–0.05	87.4
Cell-4	78.3	–0.03	88.0
**Mobile**			
Acetonitrile	59.8	–0.08	83.6
Ethanol	69.5	–0.05	85.0
Methanol	72.5	–0.04	85.9

a Standard error quartiles: enthalpies 3.3–8.9; entropies 0.01–0.03; Gibbs energies 3.3–8.9.

### Molecular Dynamics and Quantum Chemical Calculations

Activation energies were estimated via quantum chemical calculations performed in the Schrödinger Suite by evaluating energy differences between transition-state or high-energy conformations and the corresponding stable conformers of loratadine. Transition-state barriers were obtained from linear-synchronous transit (LST) calculations as the difference between the solution-phase electronic energy of the optimized transition-state structure and that of each stable conformer. In addition, activation energies were extracted from two-dimensional QM coordinate scans by identifying the maximum energy along the scanned surface within defined dihedral regions and referencing it to selected low-energy conformations. All computational activation energies represent electronic and solvation contributions and are reported in kilojoules mer mole. The LST-derived activation energies were 82 kJ mol^–1^ in acetonitrile, 96 kJ mol^–1^ in ethanol, and 209 kJ mol^–1^ in methanol, with forward and reverse barriers being effectively identical in all solvents. QM coordinate-scan barriers were substantially lower, yielding forward activation energies of 37.4, 35.7, and 36.0 kJ mol^–1^ and reverse activation energies of 27.1, 27.4, and 28.2 kJ mol^–1^ in acetonitrile, ethanol, and methanol, respectively, and exhibited only weak solvent dependence. A schematic representation of the two scanned dihedral angles and the corresponding stable conformers of loratadine is shown in Figure 8.

**Figure 8:**
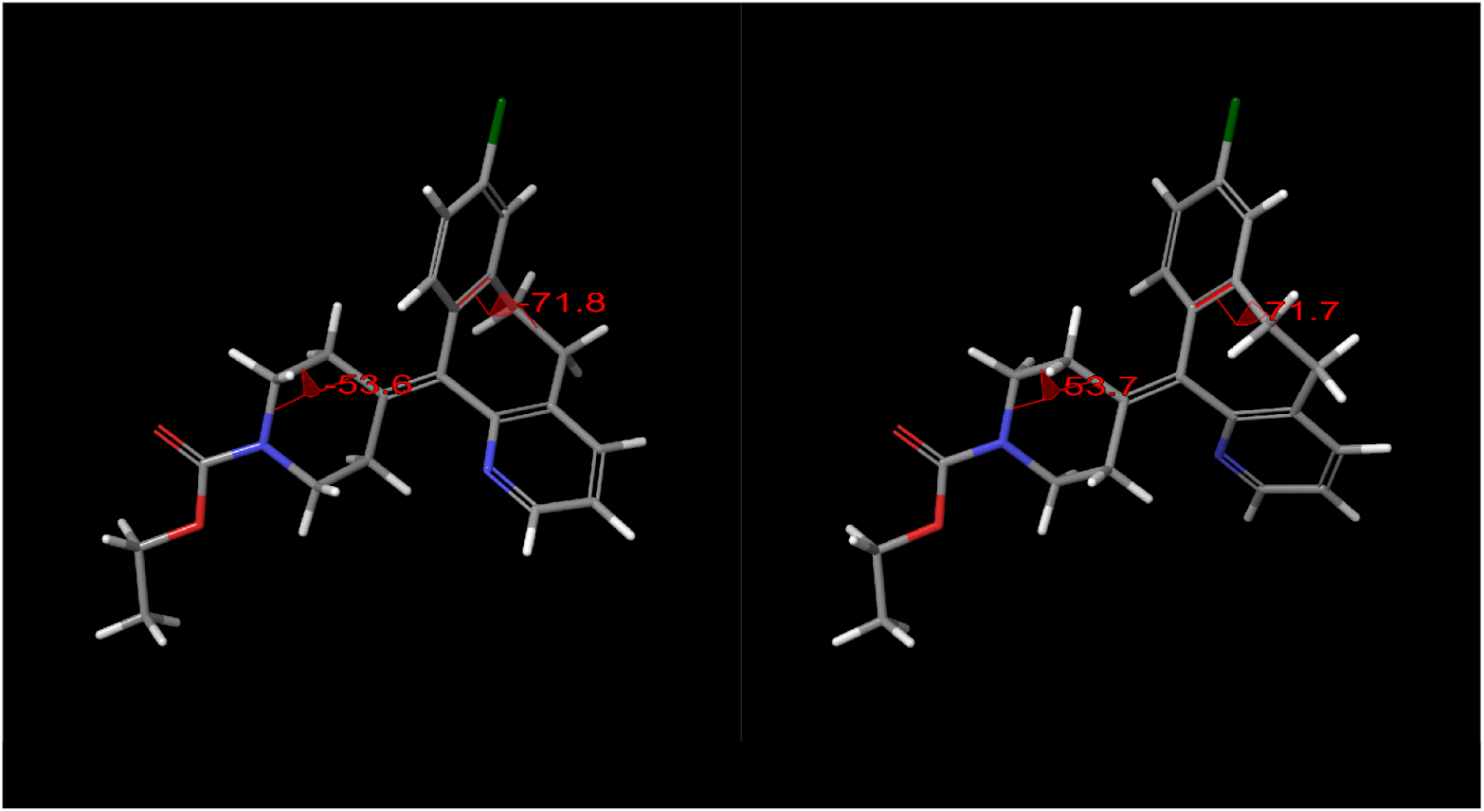
Optimized solution-phase geometries of the two stable conformers of loratadine in methanol employed in the QM coordinate-scan analysis. The two scanned dihedral angles are indicated. These conformers define the low-energy endpoints used for estimating activation energies from the two-dimensional potential energy surface. Because the conformers correspond to enantiomeric states, the dihedral angles should theoretically be exact mirror values (as described in the Methods section). However, due to the finite grid resolution used during the coordinate-scan optimization, the located minima correspond to the conformers depicted here.

## Discussion

### Two-Site Model of Chiral Chromatography

One objective of this work was to extend the automated Batman peak-fitting workflow to a two-site stochastic model, in which retention and interconversion are governed by a mixture of enantioselective and nonselective adsorption sites. In principle, this model offers a more realistic representation of chiral selectors with heterogeneous binding landscapes. In practice, however, the additional degrees of freedom substantially complicate the optimization process. Although convergence of the two-site model was formally successful even when the nonselective/enantioselective site ratio was left as a free parameter, the resulting parameter estimates were often unstable and frequently lacked physical plausibility. This behavior persisted even when stringent upper/lower bounds were imposed, indicating that the problem is structurally underdetermined in the absence of prior constraints. Consequently, *a priori* knowledge of the site ratio, or at a minimum tight restrictions informed by isotherm experiments, is required for robust two-site stochastic fitting. In practice, site ratios obtained from isotherm analysis were supplied to the fitting algorithm on a logit scale, and lower and upper bounds were constructed such that the run-specific site visitation ratio was allowed to vary within a window of ± 1 logit unit around the *a priori* estimate. Even with these safeguards, overfitting remained possible and *post hoc* inspection of parameter distributions was therefore essential to ensure interpretability. While more elaborate variants of the model could in principle accommodate site-specific interconversion kinetics or a distribution of heterogeneous sites, the rapidly increasing number of parameters would violate the principle of parsimony. For routine analysis, the added complexity of such extensions is unlikely to be justified unless independent measurements can constrain the additional parameters.

### Isotherm Analysis

The determination of the relative abundance of enantioselective and nonselective sites was achieved through overload experiments followed by fitting to a competitive bi-Langmuir isotherm. Because interconversion also occurs under overload conditions, the isotherm model relies on simplifying assumptions, most notably that the two enantiomers behave independently on the chromatographic time scale. A more rigorous future extension would incorporate interconversion explicitly into the isotherm framework, although this would substantially increase the model’s complexity.

In this study, isotherm analysis was performed at the lowest investigated temperature where the rate of interconversion was minimal. The extracted site ratios were then carried forward to higher temperatures as fixed inputs to the two-site kinetic model, an assumption that may not hold universally, especially for systems where adsorption equilibria are strongly temperature dependent. Although frontal analysis^39^ could yield more accurate site-capacity values, it is not always experimentally feasible. Nevertheless, even with its limitations, the isotherm analysis provided sufficiently consistent site ratios (in agreement with previous literature data: Table 5 in the work of Asnin 2012 ^39^) to support the kinetic decomposition.

### Phase-Specific Parameters

The major aim of this work was to demonstrate that an elaborate experimental design of chromatographic measurements, spanning several mobile phases, stationary phases, and temperatures, could be leveraged to decompose the apparent interconversion rates into mobile-phase- and stationary-phase-specific contributions. This was accomplished by formulating a mixed-effects model in which the temperature-dependent mobile-phase rate constants appear as random intercepts, while stationary-phase forward and reverse rate constants appear as random slopes with respect to a capacity factor-based transformation of the apparent kinetic constants. This decomposition implicitly assumes that mobile-phase and stationary-phase interconversion kinetics are independent, such that adsorption–desorption processes redistribute residence time between phases but do not alter the intrinsic rate constants within each phase. The assumption is reasonable under dilute conditions and for cellulose-based chiral stationary phases.

Eyring–Polányi inspection of the phase-specific parameters revealed that pooling kinetic constants from individually fitted chromatograms affords stable estimation of mobile phase-specific parameters; however, stationary phase-specific parameters, while displaying the expected temperature dependence, were considerably noisier. The Eyring–Polányi analysis also afforded phase-specific activation enthalpies and entropies for the interconversion process. The values compiled in Table 1 reveal striking differences between mobile phase and stationary phase thermodynamics. Notably, the apparent thermodynamic parameters do not always lie between their corresponding phase-specific contributions. This phenomenon, previously noted in multisite van’t Hoff analyses,^40^ may arise from model uncertainty or from true multipathway behavior that violates simplistic averaging assumptions.

A more intriguing finding concerns mobile phase-specific enthalpies: for acetonitrile, ethanol, and methanol, these values are 162 ± 34, 66 ± 9, and 69 ± 13 kJ mol^–1^, respectively. Although acetonitrile yielded the largest activation enthalpy among the eluents, it simultaneously produced the fastest interconversion, as shown both by chromatographic fits (Figure 1) and by independent CD measurements (Figure 6). This apparent contradiction is resolved upon considering Gibbs activation energies: at the median temperature (298.15 K), these values are 83 ± 34, 84 ± 9, and 84 ± 13 kJ mol^–1^. These mixed-effects model-derived values are consistent with CD-derived Gibbs activation energies (82.0 ± 0.5, 83 ± 2, and 83.7 ± 0.7 kJ mol^–1^); however, activation enthalpies from CD measurements are not in agreement (84.0 ± 0.5, 82 ± 2, and 78.0 ± 0.7 kJ mol^–1^).

Taken together, the observed disagreement among activation enthalpy estimates, the comparatively large standard errors, and the fact that column-specific thermodynamic parameters could be reliably extracted only when unified-equation kinetics were used all point to the intrinsic limits of the present decomposition strategy. In practice, run-by-run kinetic fits might contain weakly identifiable cases, which can introduce bias. When such noisy estimates are subsequently treated as “data” in a mixed-effects framework, additional statistical issues may arise even in the presence of variance weighting, including errors-in-variables (attenuation) bias, boundary or winner’s-curse effects, and overconfidence due to incomplete propagation of per-run uncertainty. These limitations are further compounded by the nonlinear transformations applied prior to mixed-effects modeling, which render the distribution of residuals non-Gaussian, as evident from diagnostic plots (Figure 5).

By contrast, empirical Bayes or *maximum-a-posteriori* approaches explicitly share information across all chromatographic runs, thereby stabilizing inference in regimes where single-run identifiability is poor. However, the less-optimal mixed-effects modeling is still necessary to provide informative priors for Bayesian inference. Bayesian hierarchical strategies provide a more robust route to phase-specific kinetic and thermodynamic parameters and more reliably target the underlying estimands. Indeed, as depicted in the bottom plots of Figure 7, activation enthalpies obtained from empirical Bayesian inference (60 ± 2, 70 ± 3, and 73 ± 3 kJ mol^–1^) exhibit substantially lower uncertainty and lie much closer to the values derived from CD measurements, although this comparison must be interpreted cautiously given that the off-column experiments are only near-identical to chromatographic conditions. Gibbs activation energies are likewise in good agreement (84 ± 2, 85 ± 3, and 86 ± 3 kJ mol^–1^), and notably appear to be considerably better identifiable from Eyring–Polányi analysis than activation enthalpies, reflecting the partial cancellation of enthalpy–entropy covariance in the Gibbs free energy. Importantly, the observed trend in eluent-specific Gibbs activation energies of interconversion is further supported by independent quantum chemical calculations, which (i) predict identical forward and reverse activation barriers and (ii) reproduce the same monotonic increase in mobile phase activation energies from acetonitrile to ethanol to methanol. This qualitative agreement across chromatographic analysis, off-column kinetics, and *ab initio* calculations provides strong cross-validating evidence for the mechanistic interpretation.

### Computationally Derived Parameters

The comparison highlights that computational barrier heights correspond most closely to experimental activation enthalpies rather than Gibbs free energies, consistent with the electronic- and solvation-level nature of the QM calculations. The close agreement between CD enthalpies and LST-derived barriers in acetonitrile suggests that in this solvent, the experimentally observed interconversion kinetics are dominated by a well-defined conformational barrier that is reasonably captured by a static transition-state description. The nearly solvent-independent Gibbs activation energies derived from CD further indicate substantial entropy-enthalpy compensation, which is not accounted for in the QM calculations. In contrast, the pronounced divergence observed in alcohol-type eluents (where LST barriers are dramatically larger than experimental values) strongly suggests that the static transition-state model overestimates the barrier in strongly hydrogen-bonding solvents. This likely reflects a breakdown of the single-pathway transition-state description due to the enhanced solvent stabilization of intermediate conformations. Taken together, these results support a model in which solvent-dependent conformational flexibility and entropy play a central role in governing interconversion kinetics with static QM barriers providing qualitative trends but not quantitative agreement for protic solvents.

## Conclusions

In this work, we extended an automated R workflow for the analysis of on-column enantiomerization by incorporating a two-site stochastic model that explicitly distinguishes nonselective and enantioselective interactions in chiral HPLC. While the two-site formulation increases the flexibility of the model, reliable parameter estimation requires prior constraints on the site-capacity ratio. Overload experiments combined with isotherm analysis provided physically meaningful estimates of site ratios, enabling stable stochastic fitting. Although the present approach assumes negligible interconversion during overload conditions, it may be extended to fully competitive–reactive isotherms in future work. A central contribution of this study is the decomposition of apparent interconversion rates into phase-specific rates by pooling information across eluents, columns, and temperatures. While mixed-effects modeling offers a convenient first-order decomposition, our results show that empirical Bayesian inference provides a more robust and statistically coherent framework. By sharing information across runs and imposing physically motivated regularization, the empirical Bayes approach stabilizes inference, reduces uncertainty, and yields phase-specific kinetic and thermodynamic parameters that are consistent with independent off-column CD measurements. Overall, this work demonstrates that conventional chiral HPLC experiments, when coupled with mechanistic peak-shape modeling, nonlinear isotherm analysis, and hierarchical inference, can yield detailed insight into enantiomerization kinetics and thermodynamics without specialized instrumentation. The workflow developed here offers an extensible framework for studying dynamic chromatographic phenomena and highlights opportunities for integrating stochastic modeling, mass-transfer theory, and modern statistical inference into next-generation analytical tools.

## Supplementary Material





## Notes

Description of the files and folders in the ZIP archive (PDF)
Additional experimental details; HPLC, overload experiments, and circular dichroism data; Batman peak fitting results; supplementary figures and tables; and code and input files required to reproduce the data analysis (ZIP)
