# Supplementary material for: Phase-Specific Parameter Estimation in Chiral HPLC Using 1‑Site, 2‑Site Stochastic Models, and Unified Equation Approach
Source: Anal Chem. 2026 Apr 29;98(18):13925–39. doi: 10.1021/acs.analchem.6c01634 (PMC13177292; doi:10.1021/acs.analchem.6c01634)
Supplement: Supplementary file 1 [file ac6c01634_si_001.pdf]

Supporting Information

Phase-Specific Parameter Estimation in Chiral HPLC Using 1-Site, 2-Site Stochastic Models and Unified Equation Approach

Arash Mirzahosseini[1,2,*] Ali Mhammad[1,2] Gergely Dombi[1,2]
Balázs Balogh[3,2] Annamária Sepsey[4] Tamás Pálla[1,2]
Simon Horváth[5] Eliza Tóth[1,2,5] Zoltán-István Szabó[6,7]
Gábor Németh[5] Attila Felinger[4,8] Oliver Trapp[9]
Gergő Tóth[1,2]

[1] Department of Pharmaceutical Chemistry, Semmelweis University, Budapest, H-1092, Hungary

[2] Center for Pharmacology and Drug Research & Development, Semmelweis University, Budapest, H-1085, Hungary

[3] Department of Organic Chemistry, Semmelweis University, Budapest, H-1092, Hungary

[4] Institute of Bioanalysis, Medical School, University of Pécs, Pécs, H-7624, Hungary

[5] Drug Substance Analytical Development Division, Egis Pharmaceuticals PLC, Budapest, H-1106, Hungary

[6] Department of Pharmaceutical Industry and Management, George Emil Palade University of Medicine, Pharmacy, Science and Technology of Targu Mures, Targu Mures, 540142, Romania

[7] Sz-imfidum Ltd., Covasna, 525401, Romania

[8] Department of Analytical and Environmental Chemistry and Szentágothai Research Center, University of Pécs, Pécs, H-7624, Hungary

[9] Department of Chemistry, Ludwig-Maximilians-Universität München, München, D-81377, Germany

[*] Correspondence: Arash Mirzahosseini <mirzahosseini.arash@semmelweis.hu>

Contents

Overview **S3**

1. Overload Experiments **S3**

2. Unified Equation, 1-Site and 2-Site Stochastic Modeling **S3**

3. Circular Dichroism Measurements **S4**

4. Kinetic and Thermodynamic Analysis with Mixed-Effects Model **S4**

5. Empirical Bayesian inference **S5**

6. Full Bayesian inference MCMC **S6**

7. Computational Chemistry **S6**

Reproducibility Notes **S7**

Overview

The Supporting Information contains all data files and analysis scripts required to reproduce the results, figures, and tables presented in the manuscript. All analyses were performed in R (version 4.5.1), and folder-level descriptions are provided below in sequence of data analysis.

When processing each folder the working directory in the script must point to its own folder.

1. Overload Experiments

- Folder: `01_isotherm_fit/`
- Script: `isotherm_analysis.R`

This folder contains raw chromatographic data from the overload experiments: `CELL1_data/`, ...

Summary tables of experimental conditions (temperature, analyte concentration, peak moments) are also provided: `CELL1_overload_data.csv`, ...

Running the script `isotherm_analysis.R` performs preprocessing of the overloaded chromatograms, followed by fitting competitive bi-Langmuir isotherm model to the peaks with highest overload for rough estimation of non-selective/enantioselective site ratio.

2. Unified Equation, 1-Site and 2-Site Stochastic Modeling

- Folders:
  - `02_Batman_fitting/`
  - `03_data_allEluents/`
- Scripts:
  - `02_Batman_fitting/Batman_v2_script.R` (contains required functions)

– 02_Batman_fitting/Batman_analysis.R (example workflow)

The **2-site version** of the stochastic BATMAN model can be run using the functions implemented in Batman_v2_script.R using site ratios extracted from previous isotherm analysis. Example code demonstrating the full analysis workflow is provided in Batman_analysis.R. The analysis script feeds in chromatograms from folder 03_data_allEluents/.

The 02_Batman_fitting/ folder also contains the exported results from both the **1-site** and **2-site** modeling approaches at each solvent level: ACN_summary_data.csv, ACN_2_summary_data.csv, ...

For details on use check: https://github.com/mirzahosseini-arash-semmelweis/Batman_chromat

3. Circular Dichroism Measurements

- Folder: 04_CD_measurements/
- Script: CD_analysis.R

The folder contains CD spectral time-series used to characterize enantiomerization behavior and temperature-dependent CD signal evolution in solvent-specific folders: ACN/, ET/, ME/. These folders contain UV absorbance and circular dichroism data tables, and experimental metadata. All analyses are performed by the script CD_analysis.R, which computes processed spectra, signal statistics, and derived thermodynamic quantities where applicable into export files:

- cd_results.csv (fitted parameters from CD measurements)
- G_data.csv (G-factors calculated from CD parameters)

4. Kinetic and Thermodynamic Analysis with Mixed-Effects Model

- Folder: 05_MEM/

- Scripts:
  - k_analysis.R
  - plot_runs.R

This script extracts kinetic and thermodynamic parameters from isotherm data, stochastic modeling results, and CD measurements. It includes routines for rate estimation, Eyring-Polányi analysis, and uncertainty quantification from the mixed-effects model. Included tables:

- k_phase_spec.csv: phase-specific kinetic parameters from mixed-effects model
- eyring_pooled.csv: apparent thermodynamic parameters
- eyring_phase_spec.csv: phase-specific thermodynamic parameters from mixed-effects model

The script plot_runs.R generates plots for a subset of representative chromatographic runs. This includes chromatograms, model fits and performance.

5. Empirical Bayesian inference

- Folder: 06_empBayes/
- Script: EB_analysis.R

This folder contains the script for Empirical Bayesian global fitting. First all chromatograms and their fitted parameters are called in from prior analyses. Preprocessing of chromatograms for empirical Bayesian inference are shown with diagnostic plots in subfolder plot_moments/. The global fitting algorithm consequently generates posterior parameters which are exported in CSV files.

Included files:

- priors.xlsx: Prior parameter estimation from mixed-effects model results
- plots_moments/: Peak moment diagnostic plots
- failed_moments.csv: Excluded runs with invalid moments
- k_spec_EB.csv: EB rate constant estimates

- therm_col_spec.csv: Column thermodynamic parameters
- therm_elu_spec.csv: Eluent thermodynamic parameters

6. Full Bayesian inference MCMC

Folder: 07_BayesianMCMC/

This folder contains the Stan code used for full Bayesian inference together with the associated input files. Although the inferred parameters were generally consistent with the empirical Bayesian results, MCMC sampling was computationally demanding and occasionally exhibited convergence issues, reflecting limitations introduced by surrogate approximations and the absence of built-in IFFT and Bessel function support in Stan. This folder contains the code and supporting files used to explore full Bayesian inference in Stan for the interconversion model. The script precompute_exchange_surrogates.R generates the file exchange_surrogates.rds, which stores polynomial surrogate mappings that approximate the Bessel-function-based exchange kernels and their sum-of-exponentials representations; exchange_model_soe.stan implements the corresponding Stan model using these surrogates for MCMC-based parameter estimation; and compile_data.R prepares the experimental peak-profile data, metadata, priors, and surrogate objects into the final stan_data list required for model fitting.

7. Computational Chemistry

- Folder: 08_compChem/
- Script: analysis.R

This folder contains the relevant files of Schrödinger suite computational chemistry calculations (inputs generated from Schrödinger: TS.csv, QM_coordscan.csv) and analysis that generates outputs: TS_results.csv, QM_results.csv.

Reproducibility Notes

- All scripts assume the folder structure as provided.
- Paths may be adjusted in the scripts if files are relocated.
- All `.csv` data files are plain text and can be opened with any standard software.
