# Supplementary material for: Phase-Specific Parameter Estimation in Chiral HPLC Using 1‑Site, 2‑Site Stochastic Models, and Unified Equation Approach
Source: Anal Chem. 2026 Apr 29;98(18):13925–39. doi: 10.1021/acs.analchem.6c01634 (PMC13177292; doi:10.1021/acs.analchem.6c01634)
Supplement: Supplementary file 2 [file ac6c01634_si_002.zip › SupportingInformation/07_BayesianMCMC/conv.pdf]

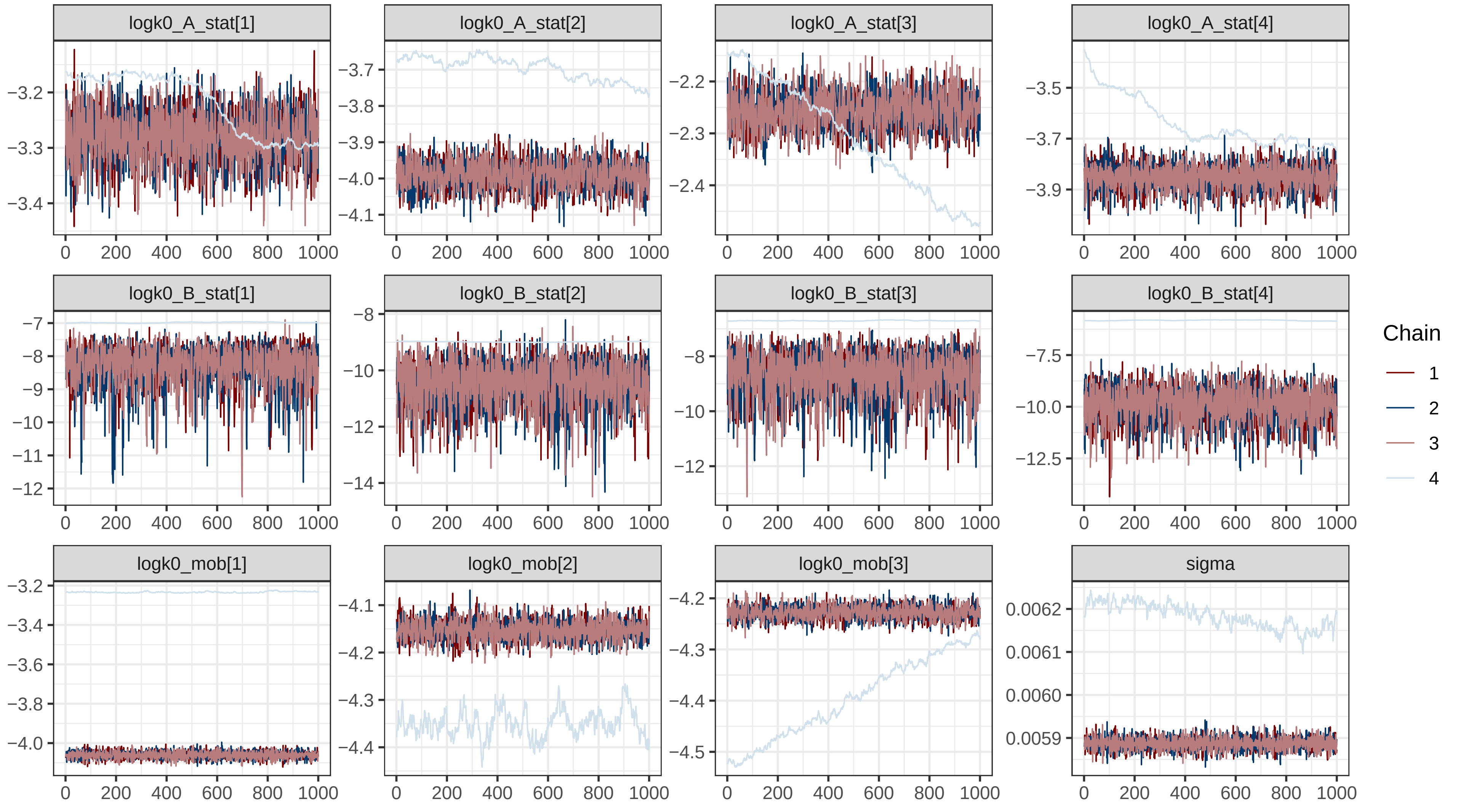

logk0_A_stat[1]
logk0_A_stat[2]
logk0_A_stat[3]
logk0_A_stat[4]
logk0_B_stat[1]
logk0_B_stat[2]
logk0_B_stat[3]
logk0_B_stat[4]
logk0_mob[1]
logk0_mob[2]
logk0_mob[3]
sigma
Chain
1
2
3
4
0 200 400 600 800 1000
