# Supplementary material for: Phase-Specific Parameter Estimation in Chiral HPLC Using 1‑Site, 2‑Site Stochastic Models, and Unified Equation Approach
Source: Anal Chem. 2026 Apr 29;98(18):13925–39. doi: 10.1021/acs.analchem.6c01634 (PMC13177292; doi:10.1021/acs.analchem.6c01634)
Supplement: Supplementary file 2 [file ac6c01634_si_002.zip › SupportingInformation/07_BayesianMCMC/pcc_plot.pdf]

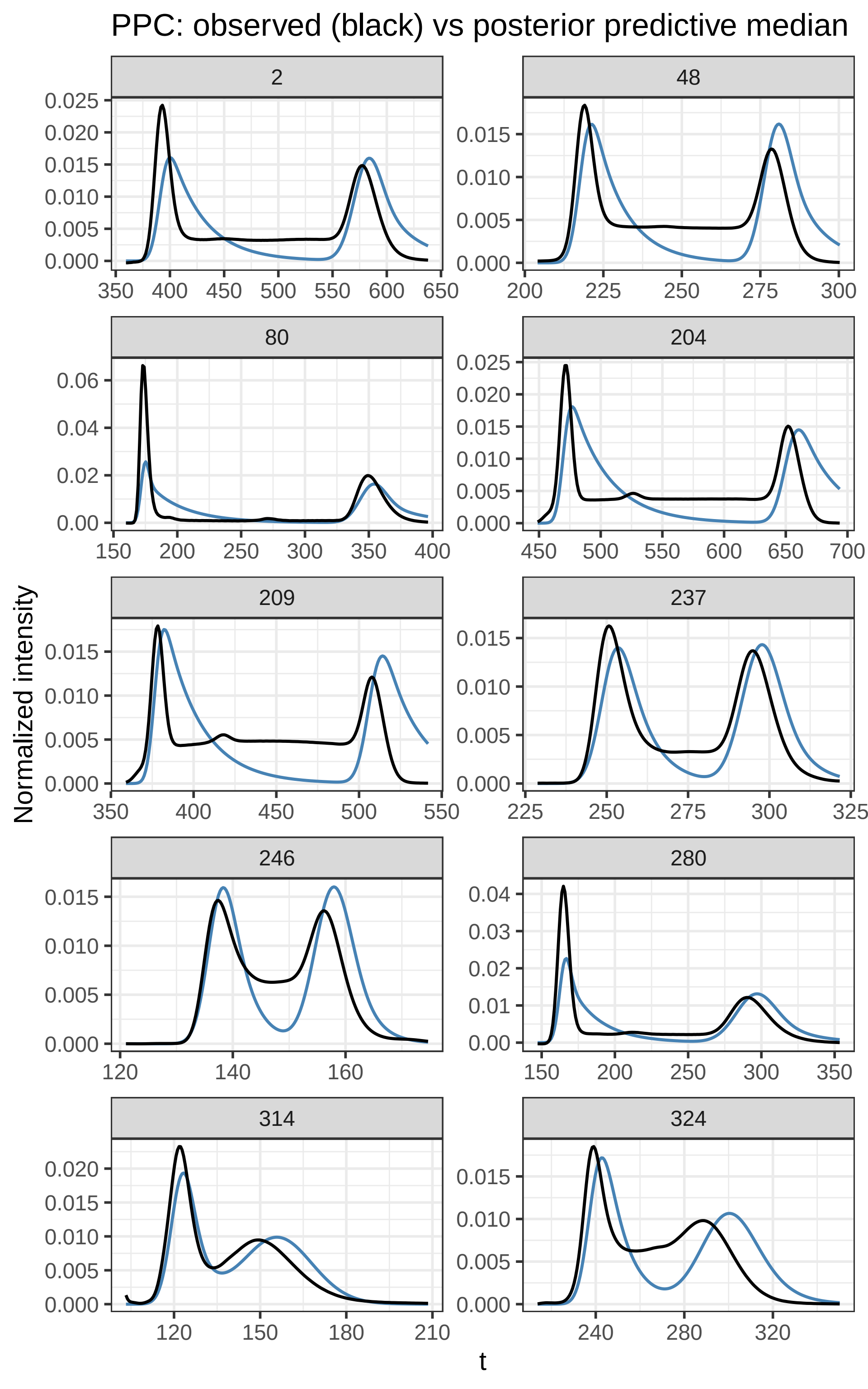

PPC: observed (black) vs posterior predictive median
2
48
80
204
209
237
246
280
314
324
Normalized intensity
t
